# Perceived Utility of Combining Atypical Long‐Acting Injectable Antipsychotics With Psychosocial Interventions: An Exploratory Survey of Mid‐Career Psychiatrists in Japan

**DOI:** 10.1002/npr2.70173

**Published:** 2026-10-06

**Authors:** Shun Umetsu, Yoshiyo Oguchi, Nobumi Miyake

**Affiliations:** ^1^ Department of Psychiatry Toyohashi Mental Health Care Center, Matsuzaki Hospital Toyohashi Aichi Japan; ^2^ Department of Psychiatry Teikyo University Hospital, Mizonokuchi Kawasaki Kanagawa Japan; ^3^ Department of Neuropsychiatry St. Marianna University School of Medicine Kawasaki Kanagawa Japan; ^4^ Department of Psychiatry Ashikaga Fujimidai Hospital Ashikaga Tochigi Japan; ^5^ Department of Neuropsychiatry Kawasaki Municipal Tama Hospital Kawasaki Kanagawa Japan

**Keywords:** attitude, Japan, long‐acting injectable, psychosocial interventions, schizophrenia

## Abstract

**Aims:**

Although combining long‐acting injectable antipsychotics (LAIs) with psychosocial treatments for schizophrenia is important, psychiatrists' attitudes toward this approach remain underexplored. We investigated Japanese psychiatrists' perceptions of combining atypical LAIs with psychosocial interventions.

**Methods:**

An anonymous cross‐sectional web‐based survey was administered to 1038 mid‐career Japanese psychiatrists, of whom 69 provided complete responses. Participants' general attitude toward recommending atypical LAIs was dichotomized as positive or negative/neutral, and they rated on a 4‐point scale the usefulness of combining atypical LAIs with five psychosocial interventions: (1) psychosocial treatment (e.g., psychoeducation), (2) day/night care, (3) community care, (4) financial/employment support, and (5) housing support.

**Results:**

Thirty‐nine respondents held a positive attitude toward recommending atypical LAIs, whereas 30 held a negative/neutral attitude. A positive attitude was significantly associated with perceiving the combination as useful for psychosocial treatment (*p* = 0.038), financial/employment support (*p* = 0.015), and housing support (*p* = 0.041), but not for day/night care (*p* = 0.090) or community care (*p* = 0.079). Free‐text rationales most commonly cited improved adherence and relapse prevention, enhanced illness insight, and synergistic effects as reasons for valuing the combination.

**Conclusions:**

This exploratory study suggests that mid‐career Japanese psychiatrists, particularly those with a favorable attitude toward atypical LAIs, perceive the combination of LAIs with psychosocial support not merely as an adherence tool but as a synergistic strategy promoting broader functional recovery. Because respondents represent a small, predominantly male, self‐selected sample, the generalizability of the findings is limited.

## Abstract 2

Among 69 mid‐career Japanese psychiatrists, a favorable attitude toward atypical long‐acting injectable antipsychotics (LAIs) was significantly associated with valuing their combination with psychosocial treatment, employment support, and housing support. LAIs may be seen as a foundation for functional recovery, not merely for adherence.

## Graphical abstract

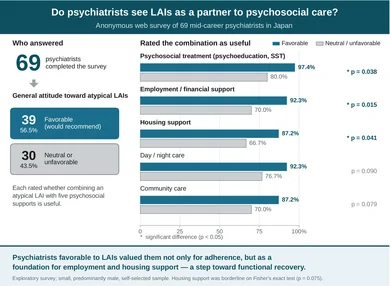

## 1 Introduction

Nonadherence to medication is a major challenge in the long‐term management of schizophrenia and is strongly associated with relapse and rehospitalization [1, 2]. Long‐acting injectable antipsychotics (LAIs), developed to address this issue, provide assured medication delivery and may improve clinical outcomes [3]. However, LAI use has remained modest in Japan; a survey of patients with schizophrenia conducted in early 2020 reported a usage rate of 16.4% [4].

In a 2011 nationwide survey conducted shortly after atypical LAIs were introduced in Japan, psychiatrists who actively recommended depot antipsychotics placed greater importance on combining LAIs with psychosocial support than those who did not [5]. In the subsequent decade, the therapeutic landscape evolved with the widespread availability of atypical LAIs and greater emphasis on functional recovery beyond symptom control [6].

Our previous study using the same sample identified persistent barriers to LAI adoption in Japan [7]. It remains unclear how Japanese psychiatrists perceive the role of atypical LAIs when integrated with psychosocial supports, including clinical programs (e.g., psychoeducation and day care), community care (e.g., visiting nursing), and functional support (e.g., employment and housing assistance). In this exploratory study, we aimed to address this gap.

## 2 Methods

### 2.1 Participants and Procedure

This anonymous cross‐sectional survey was conducted via a web‐based platform from October 2 to October 10, 2020. Recruitment was facilitated by Can do Inc. (Tokyo, Japan), through which 1038 registered psychiatrists with at least 2 years of clinical background were invited by email to participate; non‐specialists and physicians in their initial year of psychiatric residency were excluded. Sixty‐nine psychiatrists completed the survey (response rate, 6.6%). All survey items were mandatory; therefore, no data were missing, and no imputation was required. A 2000‐yen Amazon gift card was provided upon completion. Participant selection was consistent with our previous report [7].

Prospective respondents reviewed an online document describing the study's objectives, the voluntary nature of participation, and the handling of anonymity and confidentiality; their subsequent participation was treated as informed consent. The study was deemed exempt from full ethical review by the Institutional Review Board of St. Marianna University School of Medicine because it was an anonymous physician survey involving no patient intervention or identifiable personal information, a procedure consistent with our previous report using the same dataset [7]. The study was conducted in accordance with the Declaration of Helsinki. Participant demographics are presented alongside the cohorts of Fujii et al. [5] and Heres et al. [8] in Table 1.

**TABLE 1:** Demographic characteristics of participating psychiatrists in three studies.

Demographics	Japan (Oguchi et al. [7])	Japan (Fujii et al. [5])	Germany (Heres et al. [8])
Survey year	2020	2011	2005
Year of atypical LAI introduction	2009	2009	2002
Participants, *n*	69	512	246
Male, %	97.1	80.0	66.1
Institution, *n* (%)			
University	11 (15.9)	67 (13.1)	13 (5.4)
General hospital	10 (14.5)	45 (8.8)	—
Psychiatric hospital	29 (42.0)	364 (71.1)	—
Clinic	18 (26.1)	30 (5.9)	77 (31.8)
Private practice	—	—	152 (62.8)
Other	1 (1.5)	3 (0.6)	—
No answer	—	3 (0.6)	—
Age, years	49.1 ± 10.2	Male 46.9 ± 11.4 Female 41.0 ± 8.9	Male 48.7 ± 7.4 Female 46.7 ± 7.8
Experience in psychiatry, years	20.1 ± 9.7	18.0 ± 11.5	18.1 ± 7.7

*Note:* Values are shown as *n* (%) or mean ± standard deviation. The age value for the present study (Oguchi et al. [7]) was calculated for the whole sample.

Abbreviation: LAI, long‐acting injectable antipsychotic.

### 2.2 Survey Instrument

The survey instrument was adapted from a previous German study [8] and a 2011 Japanese survey [5] and collected demographic and professional data. Two domains were analyzed. First, psychiatrists' general attitude toward recommending atypical LAIs was rated on a 4‐point scale and dichotomized as a “positive attitude” (generally recommend/strongly recommend) or a “negative/neutral attitude” (do not recommend/do not recommend much). Second, respondents rated the perceived utility of combining atypical LAIs with five psychosocial interventions on a 4‐point scale (1 = disagree, 2 = disagree somewhat, 3 = agree, 4 = strongly agree), which was dichotomized as “useful” (3–4) or “not useful” (1–2): (A) psychosocial treatment (e.g., psychoeducation, SST), (B) day/night care, (C) community care (e.g., home visits), (D) financial/employment support, and (E) housing support (e.g., group homes). Respondents were also invited to provide a free‐text rationale for each rating. The complete survey instrument, including all original items and response scales, is provided as Appendix S1.

### 2.3 Statistical Analysis

Statistical analyses were performed using SPSS Version 28.0 (IBM Corp., Armonk, NY, USA). The relationship between psychiatrists' attitudes toward LAIs and the perceived utility of combination therapies was examined using the chi‐square test or, when any expected cell count was below five, Fisher's exact test; all tests were two‐sided. Odds ratios (ORs) with 95% confidence intervals (CIs) were calculated. Statistical significance was set at *p* < 0.05. Free‐text responses were summarized by grouping recurring rationales.

## 3 Results

### 3.1 Participant Characteristics

The 69 respondents (response rate, 6.6%) were predominantly male (97.1%), with a mean age of 49.1 ± 10.2 years and 20.1 ± 9.7 years of psychiatric experience. Most were in their 40s or 50s and worked mainly in psychiatric hospitals (42.0%; Table 1).

### 3.2 Perceived Utility of Combining LAIs With Psychosocial Interventions

Thirty‐nine (56.5%) had a positive attitude toward atypical LAIs, whereas 30 (43.5%) had a negative/neutral attitude. Overall, the combination was considered useful by 62 respondents (89.9%) for psychosocial treatment, 59 (85.5%) for day/night care, 55 (79.7%) for community care, 57 (82.6%) for financial/employment support, and 54 (78.3%) for housing support. As shown in Table 2, a positive attitude toward LAIs was significantly associated with considering the combination useful for psychosocial treatment (97.4% vs. 80.0%; Fisher's exact test, *p* = 0.038), financial/employment support (92.3% vs. 70.0%; χ^2^ = 5.873, df = 1, *p* = 0.015), and housing support (87.2% vs. 66.7%; χ^2^ = 4.194, df = 1, *p* = 0.041; borderline on Fisher's exact test, *p* = 0.075). No significant association was found for day/night care (92.3% vs. 76.7%; Fisher's exact test, *p* = 0.090) or community care (87.2% vs. 70.0%; χ^2^ = 3.094, df = 1, *p* = 0.079).

**TABLE 2:** Association between psychiatrists' attitude toward atypical LAIs and the perceived usefulness of combining LAIs with psychosocial interventions (*N* = 69).

Combination item	Positive attitude (*n* = 39)		Negative/neutral (*n* = 30)		χ^2^	df	*p*	OR (95% CI)
	Useful, *n* (%)	Not useful, *n* (%)	Useful, *n* (%)	Not useful, *n* (%)				
Psychosocial treatment	38 (97.4)	1 (2.6)	24 (80.0)	6 (20.0)	—	—	0.038^a^	6.81 (1.07–43.15)
Day/night care	36 (92.3)	3 (7.7)	23 (76.7)	7 (23.3)	—	—	0.090^a^	3.33 (0.85–13.11)
Community care	34 (87.2)	5 (12.8)	21 (70.0)	9 (30.0)	3.094	1	0.079	2.77 (0.85–9.02)
Financial/employment support	36 (92.3)	3 (7.7)	21 (70.0)	9 (30.0)	5.873	1	0.015	4.61 (1.21–17.53)
Housing support	34 (87.2)	5 (12.8)	20 (66.7)	10 (33.3)	4.194	1	0.041^b^	3.21 (1.00–10.33)

*Note:* Attitude toward recommending atypical LAIs was rated on a 4‐point scale and dichotomized as positive (generally/strongly recommend; *n* = 39) or negative/neutral (do not recommend/do not recommend much; *n* = 30). Each combination item was rated on a 4‐point scale and dichotomized as useful (3–4) or not useful (1–2). Odds ratios were calculated with the Haldane–Anscombe correction.

Abbreviations: CI, confidence interval; LAI, long‐acting injectable antipsychotic; OR, odds ratio.

^a^ Fisher's exact test (two‐sided) was used because the minimum expected cell count was below five (3.04 and 4.35, respectively); the Pearson chi‐square test (df = 1) was used for the remaining items, for which all expected cell counts exceeded five. All *p*‐values are two‐sided.

^b^ For housing support, the association was of borderline robustness: Fisher's exact two‐sided *p* = 0.075.

### 3.3 Free‐Text Responses

Free‐text rationales accompanied the psychosocial‐treatment item. Among the 68 substantive comments, the main rationale for endorsing the combination was improved adherence and relapse prevention (19 respondents), followed by psychoeducational benefit and enhanced illness insight (10) and synergistic or additive effects (9). One respondent commented: “By ensuring medication adherence with an LAI, the patient's condition stabilizes, making them more receptive to psychosocial programs such as SST.” Those reporting no benefit cited limited personal experience or a belief that oral medication was sufficient.

## 4 Discussion

This study examined how mid‐career Japanese psychiatrists perceive combining atypical LAIs with psychosocial support. Respondents were divided in their general attitude toward recommending atypical LAIs (56.5% positive vs. 43.5% negative/neutral), yet a large majority endorsed the usefulness of combining LAIs with psychosocial interventions across all five domains (78.3%–89.9%). A positive attitude toward LAIs was significantly associated with the perceived utility of combining them with psychosocial treatment and, most notably, with functional support such as employment and housing assistance. Conversely, no significant association was found for day/night care or community care.

Our findings suggest an evolution in clinical perspective, with LAIs viewed not merely as an adherence tool but as an enabler of real‐world recovery. The most cited rationale in our free‐text data—that LAIs create clinical stability, facilitating engagement in psychosocial therapy—supports this interpretation. This echoes the classic finding that depot antipsychotics combined with social therapy conferred a progressively lower relapse risk, whereas the depot and oral formulations alone did not differ in the first year [9]. The association with employment and housing support implies that clinicians leverage this stability to target real‐world outcomes. This is consistent with Correll et al., who reported that although LAIs can produce symptomatic remission, full functional remission often requires adjunctive psychosocial interventions [10].

The absence of a significant association for day/night care and community care is notable, given that in the 2011 survey more than 75% of psychiatrists endorsed combining depot antipsychotics with day care and with visiting care [5]. Clinicians favorable to LAIs may prioritize these agents to focus on functional recovery over intermediate programs. However, given the modest sample size (*n* = 69) and the imbalance between comparison groups, the non‐significant findings for day/night care (*p* = 0.090) and community care (*p* = 0.079) should be interpreted as trend‐level associations rather than a definitive absence of relationship. The possibility of a Type II error should therefore be considered, and the proposed “streamlined pathway” remains exploratory and hypothesis‐generating. This interpretation is nonetheless consistent with meta‐analytic evidence from Olagunju et al. [11], indicating that these agents can enhance psychosocial functioning.

However, the free‐text responses also revealed an evidence–practice gap. The notion among some clinicians that LAIs offer no benefit over oral agents is inconsistent with meta‐analytic evidence indicating a lower risk of relapse or hospitalization with LAIs [12] and with real‐world data showing better adherence and persistence with LAIs [13]. Structured communication approaches have been developed to help clinicians introduce LAIs to patients [14], and our findings underscore the need for continued education to bridge this gap.

Clinically, these findings support integrated care pathways that connect patients stabilized by LAIs to vocational and housing services, with care adjusted to each service user's goals through an equitable, multidisciplinary partnership [15].

These interpretations must be considered in light of several limitations. First, the modest sample size (*N* = 69), the low response rate (6.6%), and the imbalance between comparison groups (39 vs. 30) limit generalizability and may have reduced the statistical power to detect modest associations. Second, respondents were predominantly male (97.1%) and self‐selected; clinicians with an interest in LAIs may have been more likely to participate, which could have inflated the perceived utility reported here relative to the broader Japanese psychiatric community. Third, the cross‐sectional design precludes causal inference, and the adapted instrument was not formally validated in this sample. This study should therefore be regarded as exploratory and hypothesis‐generating.

In conclusion, in this sample of Japanese psychiatrists, atypical LAIs are viewed as a valuable adjunct to psychosocial interventions aimed at functional recovery, with clinicians favorable to LAIs particularly endorsing their combination with employment and housing support. These findings reflect the perceptions of a small, self‐selected group of mid‐career psychiatrists and should not be generalized to all psychiatrists in Japan without appropriate caution. Further studies with larger and more representative samples are required to confirm these trends.

## Author Contributions

S.U. contributed to the study concept, data analysis, and writing of the initial draft. Y.O. conceptualized and designed the study, supervised data collection and analysis, and critically revised the manuscript for important intellectual content. N.M. served as an internal reviewer and critically revised the manuscript. All the authors have read and approved the final manuscript.

## Funding

The authors have nothing to report.

## Ethics Statement

Approval of the research protocol by an institutional review board: The study was deemed exempt from ethical review by the Institutional Review Board of St. Marianna University School of Medicine.

## Consent

Participation following review of an online explanatory document was treated as informed consent (see Methods).

## Conflicts of Interest

Dr. Umetsu declares no conflicts of interest. Dr. Oguchi has received speaker honoraria from Dai‐ichi Sankyo, Eisai, Janssen, Kyowa, Otsuka, Shionogi, Sumitomo Pharma, Takeda, Lundbeck, Meiji, Mochida, MSD, and Viatris, and has received a scholarship from Eisai. Dr. Miyake has received speaker honoraria from Boehringer Ingelheim, Eisai, Meiji, Otsuka, Sumitomo Pharma, Takeda, and Viatris.

## Supporting information


**Data S1:** Raw survey data.

**Appendix S1:** Complete survey instrument (English translation [Part A] and original Japanese version [Part B]).

## Data Availability

The data supporting the findings of this study are available in Data S1 and Appendix S1 of this article.
