# Supplementary material for: Perceived Utility of Combining Atypical Long‐Acting Injectable Antipsychotics With Psychosocial Interventions: An Exploratory Survey of Mid‐Career Psychiatrists in Japan
Source: Neuropsychopharmacol Rep. 2026 Oct 6;46(4):e70173. doi: 10.1002/npr2.70173 (PMC13641931; doi:10.1002/npr2.70173)
Supplement: Supplementary file 1 [file NPR2-46-e70173-s002.docx]

＜Part A. English translation＞

**Survey on the Status and Perceptions of Atypical Long-Acting Injectable (LAI) Antipsychotics for Schizophrenia**

**(Estimated completion time: 15 minutes)**

**Introduction and Request for Cooperation**

**Objective:** Schizophrenia is a chronic and relapsing psychiatric disorder, and repeated relapses are reported to be associated with poor long-term prognosis. Therefore, preventing relapse is a critical challenge in the treatment of schizophrenia. From this perspective, improving medication adherence is considered extremely important. In reality, however, it is difficult to maintain sufficient medication adherence in all patients, and poor adherence is often a factor in symptomatic relapse or recurrence.

Long-acting injectable (LAI) antipsychotics are expected to reduce the burden of medication for patients and reduce the risk of relapse by ensuring administration. Nevertheless, a 2011 survey comparing Japan and Germany highlighted a relative lack of experience with LAIs in Japan. Currently, three atypical LAIs (hereinafter referred to as "atypical LAIs") have been launched and are expected to play a central role in LAI treatment. We have planned this survey for psychiatrists involved in the treatment of schizophrenia to re-examine the current status and challenges of atypical LAI therapy from the viewpoint of relapse prevention.

In order to investigate the issues surrounding relapse prevention in schizophrenia treatment, we kindly request your cooperation.

- This survey consists of four parts.
- This survey is conducted and aggregated entirely online.
- Please answer by circling or checking (☑) the number of the applicable item, or by entering numbers/text in the parentheses.
- All survey results will be processed statistically; individual identities and personal information will be kept strictly confidential, and you will not be inconvenienced in any way.

**Part 1: Clinician Profile**

(Please select one answer, or if multiple apply, check ☑ the one that is most applicable)

- **F1. Gender:** □ 1. Male / □ 2. Female
- **F2. Age:** ( ) years old
- **F3. Years of experience in psychiatry:** ( ) years
- **F4. Primary area of expertise:** □ 1. Clinical Psychopharmacology / □ 2. Psychotherapy / □ 3. Community Mental Health / □ 4. Forensic Psychiatry / □ 5. Child and Adolescent Psychiatry / □ 6. Other ( ) / □ 7. None in particular
- **F5. Primary disorders treated:** □ 1. Schizophrenia / □ 2. Mood Disorders / □ 3. Community Medicine / □ 4. Forensic Psychiatry / □ 5. Child and Adolescent Psychiatry / □ 6. Other ( )
- **F6. Primary clinical setting (Please circle the ward type: Open, Closed, or Forensic. Multiple selections allowed):** *Note: Forensic settings include designated medical institutions for inpatients/outpatients under the Medical Treatment and Supervision Act.* □ 1. University Hospital (Open / Closed / Forensic) □ 2. General Hospital (Open / Closed / Forensic) □ 3. Psychiatric Hospital (Single-specialty) (Open / Closed / Forensic) □ 4. Clinic □ 5. Other ( )
- **F6-1. Facility Location:** ( ) Prefecture, ( ) City/Ward/Town/Village
- **F7. Number of Patients Treated:** Please enter the approximate number of patients with schizophrenia for whom you are the primary physician, categorized by inpatient and outpatient status.
  - **Inpatient:** □ 1. None / □ 2. 0–20 / □ 3. 21–40 / □ 4. 41–60 / □ 5. 61–80 / □ 6. 81–100 / □ 7. 101 or more
  - **Outpatient:** □ 1. None / □ 2. 0–50 / □ 3. 51–100 / □ 4. 101–150 / □ 5. 151–200 / □ 6. 201 or more
- **F7-1. Ratio of Schizophrenia Patients:** Among all the patients you treat as the primary physician, what is the approximate percentage of patients with schizophrenia?
  - Inpatient (Total inpatients = 100%): ( )%
  - Outpatient (Total outpatients = 100%): ( )%
- **F8. Medication Utilization:** Regarding the schizophrenia patients you are currently treating, please enter the approximate percentage of those using atypical LAIs.
  - Inpatient (Total inpatients = 100%): ( )%
  - Outpatient (Total outpatients = 100%): ( )%
- **F9. Stance on Explanation:** When explaining atypical LAIs to patients, which of the following best describes your clinical stance?
  - **Question:** Do you strongly persuade and actively recommend by saying, "I think you need an atypical LAI"?
  - **Options:** 1. Do not recommend / 2. Do not recommend much / 3. Generally recommend / 4. Strongly recommend/Actively persuade

**Part 2: Questions Regarding Atypical LAIs**

The following questions (Q.) are the Japanese version (shortened version) of a survey conducted in Germany by Dr. W. Kissling and Dr. S. Heres. The purpose is to identify challenges regarding LAIs and to compare perceptions with the 2011 survey.

**Q. Among patients with schizophrenia or schizoaffective disorder currently receiving antipsychotic treatment, some are likely not using atypical LAIs. Please rate the frequency of the following reasons for not using atypical LAIs on a 5-point scale.** (1. Strongly disagree / 2. Disagree / 3. Neither/Sometimes / 4. Agree / 5. Strongly agree)

- **A)** Sufficient medication adherence is obtained with oral medications.
- **B)** In principle, atypical LAIs are not used for first-episode patients.
- **C)** Atypical LAIs have more side effects than oral medications.
- **D)** Switching from oral administration to atypical LAI is too complex.
- **E)** I have almost no clinical experience with atypical LAIs.
- **F)** The patient is concerned about the pain of the injection.
- **G)** I did not recommend an atypical LAI at first because I assumed the patient would likely refuse it.
- **H)** The cost of atypical LAIs is too high.
- ***If there are any other reasons for not prescribing atypical LAIs, please specify below:* ( )**

**Part 3: Combination of Atypical LAIs and Psychosocial Treatment**

The following questions (Q.) are the Japanese version (shortened and modified version) of a survey conducted in Germany by Dr. W. Kissling and Dr. S. Heres. These questions are for physicians who currently prescribe atypical LAIs.

**Q. Please provide your opinion on the effectiveness of combining atypical LAIs in the following scenarios. (Please select one answer that you consider most applicable.)** (1. Disagree / 2. Disagree somewhat / 3. Agree / 4. Strongly agree)

- **A) The combination of psychosocial treatment (psychoeducation, family education, SST, etc.) and atypical LAI is effective.**
  - Score: (1 / 2 / 3 / 4)
  - **Please state the reason for your judgment:** ( )
- **B) The combination of day-care/night-care and atypical LAI is effective.**
  - Score: (1 / 2 / 3 / 4)
  - **Please state the reason for your judgment:** ( )
- **C) The combination of community care (e.g., home-visit medical care, home-visit nursing, etc.) and atypical LAI is effective.**
  - Score: (1 / 2 / 3 / 4)
  - **Please state the reason for your judgment:** ( )
- **D) The combination of economic support (employment support, etc.) and atypical LAI is effective.**
  - Score: (1 / 2 / 3 / 4)
  - **Please state the reason for your judgment:** ( )
- **E) The combination of housing support (group homes, etc.) and atypical LAI is effective.**
  - Score: (1 / 2 / 3 / 4)
  - **Please state the reason for your judgment:** ( )

**Part 4: Questions Regarding Atypical LAIs during the COVID-19 Pandemic**

Based on your experience during the COVID-19 pandemic, we would like to ask about the necessity of telemedicine and atypical LAIs in preventing relapse/recurrence in patients with schizophrenia or schizoaffective disorder.

**Q. Please provide your opinion on the following items under the spread of COVID-19.**

- **A) Compared to pre-pandemic times, what is the incidence of relapse/recurrence in the poor-adherence group?**
  1. Decrease / 2. Decrease slightly / 3. No change / 4. Increase slightly / 5. Increase
- **B) What is your opinion on telemedicine (e.g., telephone/online consultations)?**
  1. Problematic / 2. Somewhat problematic / 3. Neither / 4. Somewhat useful / 5. Useful
- **C) What is your opinion on the frequency of opportunities for telemedicine?**
  1. Decrease / 2. Decrease slightly / 3. No change / 4. Increase slightly / 5. Increase
- **D) What is your opinion on the necessity of atypical LAI treatment?**
  1. Not necessary / 2. Almost unnecessary / 3. Neither / 4. Somewhat necessary / 5. Necessary
- **E) What is your opinion on the necessity of the 3-month formulation atypical LAI (scheduled to be launched in Japan)?**
  1. Not necessary / 2. Almost unnecessary / 3. Neither / 4. Somewhat necessary / 5. Necessary

**This concludes the survey. Thank you very much for your cooperation.**

＜Part B. Original Japanese version＞

統合失調症に対する非定型抗精神病薬の

持効性注射製剤（LAI）に関する意向調査

（15分程度で回答可能です。）

【ご回答に際してのお願い】

目的：統合失調症は、慢性・再発性の精神疾患であり、再発を繰り返すことが長期的な予後不良に関連すると言われています。したがって、統合失調症治療において、いかに再発を防ぐかということが重要な課題であり、その観点から、服薬アドヒアランスを高めることが極めて重要であると考えられます。しかしながら、現実には、すべての患者において十分な服薬アドヒアランスが保たれているとは言い難く、それが症状の再発・再燃の要因となっている場合が多いと考えられます。持効性抗精神病薬注射製剤（以下LAI; long-acting injection）は、患者の服薬の負担が軽減でき、確実な投薬を可能にすることで再発リスクの軽減が期待できると言われていますが、2011年にわが国で行われたドイツと比較した調査によると、LAIの使用経験の少なさが明らかになっています。現在、非定型抗精神病薬のLAI（以下非定型LAI）が3剤上市され、LAI治療の中軸が担われていると考えられる中、改めて再発予防の観点から非定型LAI治療における現状と課題を見出すべく、統合失調症治療に携わる精神科医の先生を対象にアンケート調査を計画いたしました。

統合失調症治療における再発予防の問題を検討するために、何卒、先生方のご協力を賜りますようお願い申し上げます。

・アンケートは全部で4つのパートに分かれています。

・今回のアンケートはすべてWeb上、オンラインでの集計となります。

・ご回答の記入は該当の項目の数字に1つ○印かレ点をつける、もしくは数字や文章を記入してください。

・今回のアンケート結果は、全てコンピューター処理を実施し、統計資料として

まとめますので、先生方の個人情報の漏洩等、ご迷惑をおかけすることは一切

ございません。

1. 先生ご自身のことに関しまして、下記の項目についてお答えくださいますようお願いいたします。

（回答は1つ/複数ある場合、その中で最もだと思われる選択肢に☑）

F1. 先生の性別　　　　　　　　　□1. 男性 　　□2. 女性

F2. 先生のご年齢　　　　　　　　（　　　　）歳

F3. 先生の精神科経験年数　　　　（　　　　）年

F4. 先生の主要な専門領域

□1. 臨床精神薬理　□2. 精神療法　□3. 地域医療　□4. 司法精神医学

□5. 児童思春期　　□6. その他 （　　　　　　 ） □7. 特にない

F5. 先生が主として診療されている疾患

□1. 統合失調症　□2. 気分障害　□3. 地域医療　□4. 司法精神医学

□5. 児童思春期　□6. その他 （　　　　　　 ）

F6. 先生が主として診療されている施設（開放病棟or閉鎖病棟、触法関連施設に該当するか○をお願いいたします。複数選択可です。）

　※触法関連施設とは指定入通院医療機関等を含みます。

□1. 大学病院　　　　　開放　閉鎖　触法関連

□2. 総合病院　　　　　開放　閉鎖　触法関連

□3. 精神科病院（単科）開放　閉鎖　触法関連

□4. クリニック

□5. その他 　（　　　　　　 　　　　　　　　）

F6-1. その施設の所在地

（　　　　　　 ）都・道・府・県　　（　　　　　　 ）市・区・町・村

F7. 治療患者数

現在、その施設で先生が主治医として治療している統合失調症患者さんの人数を、入院・外来別におおよその人数でご記入ください。

|  | **入院** | **外来** |
| --- | --- | --- |
| 統合失調症治療患者さんの人数 | □1. 担当なし  □2. 0〜20人  □3. 21〜40人  □4. 41〜60人  □5. 61〜80人  □6. 81〜100人  □7. 101人以上 | □1. 担当なし  □2. 0〜50人  □3. 51〜100人  □4. 101〜150人  □5. 151〜200人  □6. 201人以上 |

F7-1. 先生が主治医として治療しているすべての患者さんの内で、統合失調症患者さんはどれくらい占めていますか。入院・外来別におおよその人数でご記入ください。

| **すべての治療患者さんのうち、統合失調症患者さんの比率は** | **入院患者を100%として**  **（　　　　　　）%** | **外来患者を100%として**  **（　　　　　　）%** |
| --- | --- | --- |

F8. 使用薬剤：現在治療している統合失調症患者さんについて、非定型LAIのおおよその割合をご記入ください

|  | **入院患者さんを**  **100%として** | **外来患者さんを**  **100%として** |
| --- | --- | --- |
| 非定型LAI | （　　　　　　）% | （　　　　　　）% |

F9. 患者さんに非定型LAIの説明をする際、主治医として下記のいずれのスタンスで対応することが多いですか。該当する対応を選択してください。

|  | **勧めない** | **それほど**  **勧めない** | **だいたい**  **勧める** | **勧める** |
| --- | --- | --- | --- | --- |
| あなたには非定型LAIが必要だと思います、と強く説得して積極的に勧めますか？ | 1 | 2 | 3 | 4 |

2. 非定型LAIに関する質問項目：

以下に示すQ.はドイツで行われたアンケート調査（Dr. W. KisslingおよびDr. S. Heres実施版）の日本語版（短縮版）です。このアンケート調査でLAIに関する問題点を確認するとともに、LAIへの認識の違いを2011年の同様の調査とで比較検討することを目的としています。

Q. 現在、抗精神病薬治療をしている統合失調症患者さんおよび統合失調感情障害患者さんのうち、非定型LAIを使用していない患者さんがいると思いますが、その患者さんに非定型LAIを使用しない理由の頻度を、5段階でお答えください。（回答は1つです。最もだと思われる選択肢に○をつけてください。）

A）経口薬に対して患者の十分な服薬アドヒアランスが得られている

　1. 全くそう思わない

　2. あまりそう思わない

　3. ときどき

　4. ほぼそう思う

　5. 強くそう思う

B）原則として非定型LAIは初発患者には使用しない

　1. 全くそう思わない

　2. あまりそう思わない

　3. ときどき

　4. ほぼそう思う

　5. 強くそう思う

C）経口薬より非定型LAIは副作用が多い

　1. 全くそう思わない

　2. あまりそう思わない

　3. ときどき

　4. ほぼそう思う

　5. 強くそう思う

D）経口投与から非定型LAIへの切り替えが複雑すぎる

　1. 全くそう思わない

　2. あまりそう思わない

　3. ときどき

　4. ほぼそう思う

　5. 強くそう思う

E）非定型LAIによる診療経験がほとんどない

　1. 全くそう思わない

　2. あまりそう思わない

　3. ときどき

　4. ほぼそう思う

　5. 強くそう思う

F）患者が注射の痛みを心配している

　1. 全くそう思わない

　2. あまりそう思わない

　3. ときどき

　4. ほぼそう思う

　5. 強くそう思う

G）おそらく患者が拒否すると思ったため、最初に非定型LAIを全く勧めなかった

　1. 全くそう思わない

　2. あまりそう思わない

　3. ときどき

　4. ほぼそう思う

　5. 強くそう思う

H）非定型LAIの費用が高すぎる

　1. 全くそう思わない

　2. あまりそう思わない

　3. ときどき

　4. ほぼそう思う

　5. 強くそう思う

※上記以外に非定型LAIを処方しない理由があれば次にお書き下さい。

3. 非定型LAIと心理社会的治療の併用に関する質問：

以下に示すQ.はドイツで行われたアンケート調査（Dr. W. KisslingおよびDr. S. Heres実施版）の日本語版（短縮改変版）で、非定型LAIを使用されている先生を対象として質問させていただきます。

Q. 下記のような場面を想定した場合に、非定型LAIとの組み合わせについて、先生のお考えをお聞かせください。（回答は1つです。最もだと思われる選択肢に○をつけてください。）

A）心理社会的治療（心理教育、家族教育、SST等）の導入と非定型LAIとの組み合わせは効果的である

　1. そう思わない

　2. あまりそう思わない

　3. そう思う

　4. 非常にそう思う

なぜ上記のように判断したのか、理由を書いて下さい

B）デイケア・ナイトケアと非定型LAIとの組み合わせは効果的である

　1. そう思わない

　2. あまりそう思わない

　3. そう思う

　4. 非常にそう思う

なぜ上記のように判断したのか、理由を書いて下さい

　　　　　　　　　　　　　　　　　　　　　　　　　　　　　　　　　　　　　　　　　　C）地域ケア（例. 訪問診療や訪問看護等）と非定型LAIとの組み合わせは効果的である

　1. そう思わない

　2. あまりそう思わない

　3. そう思う

　4. 非常にそう思う

なぜ上記のように判断したのか、理由を書いて下さい

D）経済的問題の支援（就労支援等）と非定型LAIとの組み合わせは効果的である

　1. そう思わない

　2. あまりそう思わない

　3. そう思う

　4. 非常にそう思う

なぜ上記のように判断したのか、理由を書いて下さい

E）住居支援（グループホーム等）と非定型LAIとの組み合わせは効果的である

　1. そう思わない

　2. あまりそう思わない

　3. そう思う

　4. 非常にそう思う

なぜ上記のように判断したのか、理由を書いて下さい

4. 新型コロナウイルス（COVID-19）によるパンデミック経験下における非定型LAI等に関する質問項目：

COVID-19によるパンデミックを経験した中で、統合失調症患者さんおよび統合失調感情障害患者さんの症状再燃・再発予防における遠隔診療ならびに非定型LAIの必要性についておたずねいたします。

Q. COVID-19蔓延下において、以下の設問に関する先生のお考えをお聞かせください。（回答は1つです。最もだと思われる選択肢に○をつけてください。）

A）服薬アドヒアランス不良群において通常時より症状の再燃・再発例がどうなると思われますか

　1. 減る

　2. やや減る

　3. 変わらない

　4. やや増える

　5. 増える

B）電話再診・オンライン診療等の遠隔診療についてどう思いますか

　1. 問題がある

　2. やや問題がある

　3. どちらでもない

　4. やや有用である

　5. 有用である

C）電話再診・オンライン診療等の遠隔診療の機会についてどう思いますか

　1. 減る

　2. やや減る

　3. 変わらない

　4. やや増える

　5. 増える

D）非定型LAIによる治療の必要性についてどう思いますか

　1. 必要がない

　2. ほぼ必要がない

　3. どちらでもない

　4. やや必要がある

　5. 必要がある

E）わが国で上市予定の非定型LAI 3ヶ月製剤の必要性についてどう思いますか

　1. 必要がない

　2. ほぼ必要がない

　3. どちらでもない

　4. やや必要がある

　5. 必要がある

以上、アンケートは終了です。ご多忙のところ、ご協力いただきまして誠にありがとうございました。
